# Extrapulmonary and Drug-Resistant Childhood Tuberculosis: Unveiling the Disease to Adopt the Optimal Treatment Strategy

**DOI:** 10.3390/pathogens12121439

**Published:** 2023-12-12

**Authors:** Domenico Pace, Francesca Corvaglia, Catiuscia Lisi, Luisa Galli, Elena Chiappini

**Affiliations:** Infectious Disease Unit, Department of Health Sciences, Meyer Children’s Hospital IRCCS, University of Florence, 50121 Florence, Italy; domenico.pace1@edu.unifi.it (D.P.);

**Keywords:** tuberculosis, children, extrapulmonary tuberculosis, drug resistance, therapy, second-line drugs

## Abstract

Paediatric tuberculosis (TB) is a substantial threat among infectious diseases, particularly considering the high risk of extrapulmonary tuberculosis (EPTB), severe forms of the disease, and the spreading of drug-resistant strains. Describing the characteristics of children with EPTB and those with drug-resistant tuberculosis (DR-TB) and analysing the role of second-line drugs could facilitate the management of these cases. This retrospective study was conducted on 271 children diagnosed with active TB disease (44 EPTB cases, 9 DR-TB cases), originating from diverse geographic areas, who were referred to the infectious disease unit at Meyer Children’s Hospital, Florence, Italy, from 2006 to 2022. In most patients, the management of therapies was complicated by the impossibility to obtain drug susceptibility testing (DST) results, which improved over the years: 17/154 (11.04%) children had DST results between 2006 and 2013, and 50/117 (42.73%, *p* < 0.001) between 2014 and 2022. Second-line drugs were not exclusively administered to DR-TB cases, but also to EPTB cases (20/44, 45.45%). Drugs were generally well tolerated; adverse events occurred in 13 children (13/271, 4.80%) and were generally mild and reversable. Therapies were successful in 267 children (98.52%) considered cured, while 4 (1.48%) presented sequelae. Both univariate and multivariate logistic regression analyses were conducted to investigate factors associated with EPTB, DR-TB, and second-line drugs administration. Originating from Asia emerged as a risk factor associated with both EPTB and DR-TB (*p* = 0.013 and *p* = 0.045, respectively). The introduction of GeneXpert tests has significantly improved TB diagnosis and the obtaining of DST results. The administration of second-line therapies should be limited primarily to DR-TB cases, but it is possible that these drugs may also be beneficial in selected EPTB cases.

## 1. Introduction

Historically, tuberculosis (TB) is considered one of the most significant threats in the field of infectious diseases [1]. Even though the introduction of anti-TB drugs in the mid-20th century marked a crucial moment in the management of the disease [1], TB remains a global threat today: paediatric TB accounted for 1.2 million cases and 220,000 deaths in 2021 [2]. 

Infected children present a higher risk of developing TB disease compared to adults. However, diagnosing paediatric TB can be challenging since most children exhibit non-specific signs and symptoms [3]. Moreover, the majority of cases present with paucibacillary disease, reducing the sensitivity of microbiological investigations [4]. 

Compared to the adult population, a higher proportion of children present with extrapulmonary TB (EPTB), probably resulting from the immaturity of the immune system [5]. EPTB prevalence among children remains significant, even with the availability of the Bacillus Calmette–Guerin (BCG) vaccine [2]. Moreover, EPTB is often associated with origins in Sub-Saharan Africa or Asia [6,7], and poverty stands out among major factors influencing its prevalence [8]. Severe forms such as tuberculous meningitis (TBM) and miliary TB can develop rapidly and need to be diagnosed and treated without delay, given the high risk of sequelae and death [3,9].

Drug-resistant TB is a common concern, favoured by incorrect treatment and weak medical systems [10], making the evaluation of drug susceptibility testing (DST) of each patient and contact case necessary [4] to ensure the prescription of the appropriate therapy [8]. Notably, this is difficult to obtain in children, since paucibacillary disease is associated with low sensitivity of DST [4].

TB treatment requires the administration of multiple drugs [11]. First-line drugs, including isoniazid, rifampicin, pyrazinamide, and ethambutol, exhibit significant antitubercular activity [12,13,14,15]. These drugs are administered in the treatment of drug-susceptible (DS-TB) pulmonary cases [4,9], and they are mostly associated with favourable outcomes in these patients (88% of treated children in 2020) [2]. Drug-resistant TB (DR-TB) treatment requires the administration of second-line drugs, such as levofloxacin, moxifloxacin, linezolid, clofazimine, cycloserine, amikacin and the recently approved bedaquiline, and delamanid [4,16,17]. DST results must be considered when treating DR-TB cases, and children may undergo longer individualised regimens or the shorter all-oral bedaquiline-containing regimen (if they meet criteria outlined in guidelines) [4,16,17]. Second-line drugs are combined with first-line drugs, especially in isoniazid-resistant TB treatment and in the shorter all-oral bedaquiline-containing regimen. For the administration of any first-line drug to these patients, DST results must confirm susceptibility to them [4,16,17]. The role of these drugs has been demonstrated both in vitro [18] and in vivo [19].

The treatment regimens for drug-susceptible EPTB cases depend on the affected site. Tubercular lymphadenitis cases are treated following the same regimens adopted for PTB [4]. However, managing EPTB cases is more challenging when specific sites are affected, such as the central nervous system (CNS) and osteoarticular system, or in generalised forms of the disease (miliary TB). Considering the risk of sequelae and the suboptimal distribution of drugs in these sites, guidelines suggest longer regimens for these children [4]. Despite the administration of therapies, these forms exhibit a high rate of unfavourable outcomes: TBM is associated to a mortality rate of 19.3% of cases, and only 36.7% of children survive without neurological sequelae [20].

Studies focussing on paediatric management of EPTB and DR-TB cases are not as numerous as studies on adult cases. Moreover, most studies evaluate children in low-resource countries. Considering this and the challenges that paediatric TB presents, this study intends to analyse the characteristics and the management (including diagnosis, DST evaluation, administered drugs, adherence to treatment regimens, adverse events issues, and therapies outcome) of children diagnosed with active TB originating from diverse geographic areas and admitted to the infectious disease unit (IDU) at Anna Meyer Children’s University Hospital in Florence, Italy, a tertiary referral hospital, over a period of 17 years (2006–2022). The aim of the study is to evaluate risk factors associated with DR-TB and EPTB, and to evaluate retrospectively the management and outcome of active TB in these children.

## 2. Materials and Methods

### 2.1. Definitions

Children were classified into three categories, based on the guidelines of the American Academy of Pediatrics (AAP): uninfected children, children with tuberculosis infection (TBI), and children with TB disease [16]. The study analysed children with TB disease.

TB disease was diagnosed in children with symptoms, signs, or radiographic manifestations consistent with TB disease. Diagnosis was microbiologically confirmed through laboratory detection of *M. tuberculosis* using culture, microscopy, or molecular methods from specimens of sputum, gastric aspirate, bronchial washing, pleural fluid, cerebrospinal fluid (CSF), urine, other bodily fluid, or tissue biopsy [4,16]. Children were diagnosed with TBI if they had a positive TST or IGRA result, no symptoms or signs of disease, and normal chest radiograph findings or evidence of healed infection [16].

Disease was considered DR-TB if resistant to any drug used to treat DS-TB, multidrug-resistant TB (MDR-TB) if resistant to rifampicin and isoniazid, pre-extensively drug-resistant TB (pre-XDR-TB) if resistant to rifampicin and isoniazid and to any fluoroquinolone, extensively drug-resistant TB (XDR-TB) if concomitantly resistant to rifampicin, isoniazid, at least one fluoroquinolone, and at least one of the following parenteral drugs: amikacin, kanamycin, or capreomycin [16,17,21]. Cases without drug susceptibility testing results were categorised as neither susceptible nor resistant.

### 2.2. Study Design and Population

This single-centre study included children (aged 0–18 years) diagnosed with active TB, admitted to the IDU at Anna Meyer Children’s University Hospital in Florence, Italy, between January 2006 and October 2022. 

Patient data were collected from clinical records and entered into an electronic database, including the following information: personal information (gender, date of birth, country of birth, and parents’ nationality); medical history [Bacillus Calmette–Guerin (BCG) vaccination status, HIV infection diagnosis, reason for TB investigation, symptoms, nationality, treatment, and resistance profile of the contact case]; patient management and tests conducted [dates of first and last observation, results of tuberculin skin test (TST) and IGRAs (Elispot, QuantiFERON in tube, QuantiFERON TB gold); results of microbiological investigations (microscopic examination, cultures, PCR GeneXpert, PCR GeneXpert Ultra), resistance profile, and imaging results]; treatment information (regimen and duration, adverse events, date and reason for discontinuation of drugs, and treatment outcome).

Patients and their families were asked about adverse reactions to antitubercular drugs. Considering the children’s conditions at the end of the follow-up (its duration was not standardised), cases were classified into the following outcome groups: treatment success (if cured and if the treatment was completed), treatment failure, death, lost to follow-up [22].

### 2.3. Laboratory and Microbiological Investigations

Trained nurses performed the TST using *Mantoux*’s procedure, which involves injecting 5 tuberculin units (in 0.1 mL) of purified protein derivative (Statens Serum Institute, Copenhagen, Denmark) into the volar surface of the forearm. After 48–72 h, a paediatrician from the IDU measured the resulting dermal induration. Following AAP guidelines, an induration greater than 5 mm was considered positive in children in close contact with known or suspected contagious individuals with TB disease, and in children suspected of having TB disease (with clinical or radiographic evidence). An induration greater than 10 mm was considered positive in children younger than 4 years of age, children with other medical conditions, those born in high-prevalence regions, and those frequently exposed to adults who were living with HIV infection, experiencing homelessness, incarcerated, injecting drugs, or having alcohol use disorders. All other cases were considered positive if the induration was greater than 15 mm [16].

All laboratory tests were conducted in the same laboratories at the University of Florence, following routinely performed procedures and the manufacturer’s instructions. The QuantiFERON Gold-In-Tube (QFT G-IT, Cellestis, Chadstone, VIC, Australia) was used for the IGRA testing. The QFT assays were conducted following the manufacturer’s instructions. The test result was interpreted by subtracting the value from the negative control and was considered positive if the antigen-dependent response was equal to or greater than 0.35 IU/mL, and negative if the mitogen-induced response was equal to or greater than 0.5 IU/mL, and the antigen-dependent response was less than 0.35 IU/mL. It was considered indeterminate if both the mitogen-induced and antigen-dependent responses were below the cut-off or if the mitogen-induced response exceeded 8 IU/mL [23]. 

Microscopic examination for acid-alcohol-resistant bacilli was performed using fluorescence microscopy, where the specimen is stained with auramine–rhodamine. The research of *M. tuberculosis* complex DNA was conducted using GeneXpert Ultra. During the period examined, different commercial methods were employed (Cobas–Roche, Artus Quiagen, GeneXpert MTB/RIF Ultra-Cepheid). Cultural examination was carried out on solid medium (Lowenstein Jensen), with an incubation period of 60 days, and on the liquid medium *Mycobacteria Growth Indicator Tube* (MGIT) (Becton, Dickinson, and Company), with an incubation period of 42 days. Strain identification was performed using *Line Probe Assay* (Hain), and DST was conducted using the BD MGIT™ TBc *Identification Test*. Resistance of *M. tuberculosis* was studied through DST on clinical samples, or through molecular investigation for genotypic resistance (GeneXpert Ultra). Clinical samples were collected on three consecutive days and analysed through microscopic examination, culture, and GeneXpert Ultra analysis.

### 2.4. Treatment

Patients were treated according to treatment regimens recommended by international guidelines, in particular referring to the AAP guidelines outlined in the “Red Book: 2021 Report of the Committee on the Infectious Diseases” [16]. 

### 2.5. Statistical Methods

Median and interquartile ranges (IQRs) were calculated for continuous variables (i.e., age and duration of treatment). Categorical data were compared using χ^2^ tests, or the Fisher exact test, as appropriate. Univariate logistic regression analysis was performed to investigate possible risk factors for confirmed DR-TB, and ORs with 95% confidence intervals (CIs) were calculated. Univariate and multivariate logistic regression analyses were used to investigate possible risk factors for EPTB, and to investigate factors associated with second-line drugs administration. Factors were included in the multivariate analysis if they were significant (*p* < 0.05) in the univariate analysis. Stata Software, version 14.0 (StataLLC, College Station, TX, USA) for Windows was used to perform the analyses.

## 3. Results

The study conducted an evaluation of 271 children diagnosed with TB disease (median age 6 years, IQR 3–11). Their characteristics are summarised in Table 1, Table A1 and Table A2.

### 3.1. Characteristics of the Study Population

The characteristics of patients aged ≤5 years and >5 years were similar (Table 1). Moreover, when evaluating male and female children, significant differences were not detected. 

**Figure 1:**
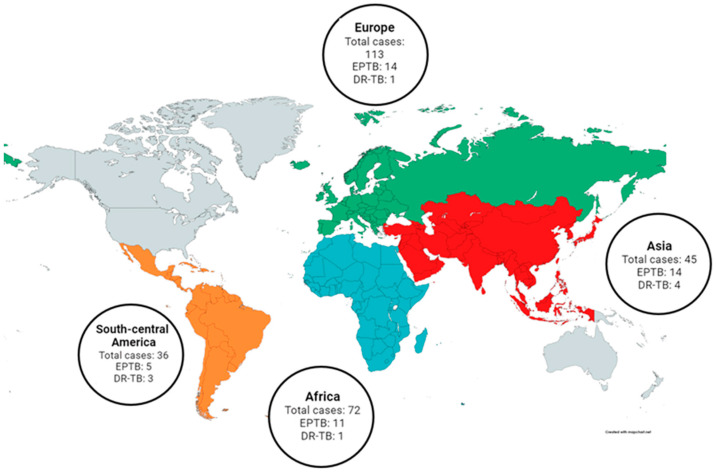
Distribution of cases (n = 271) by origin. For each continent, the number of total cases, extrapulmonary tuberculosis (EPTB) cases, and drug-resistant tuberculosis (DR-TB) cases are indicated. Created with mapchart.net.

The majority of patients were of foreign origins (Figure 1). In fact, 63 cases (23.25%) were Italian, while 208 (76.75%) were either born abroad or born in Italy to foreign parents. Among the latter group, 45 (16.60%) originated from Asia, 48 (17.71%) from Eastern Europe, 2 (0.73%) from Western Europe, 72 (26.57%) from Africa, and 36 (13.28%) from South-Central America.

These patients underwent TB disease assessment for various reasons: 144 children (53.14%) had come into contact with a diagnosed or suspected case; 99 cases (36.53%) presented with symptoms indicative of TB-disease; and 25 patients (9.22%) were identified through a screening programme directed at immigrated and adopted children. Notably, children aged ≤5 years old were mainly evaluated for TB after coming into contact with a case (86/131; 65.65%; *p* < 0.001), while children aged >5 years old primarily presented with symptoms (68/140; 48.57%; *p* = 0.001). Furthermore, there is a correlation between the reason for investigation and the type of TB-disease: PTB cases were predominantly diagnosed after coming into contact with a case (138/227; 60.79%; *p* < 0.001), while EPTB patients were more often identified after presenting with symptoms (36/44; 81.82%; *p* < 0.001).

### 3.2. Clinical Characteristics

**Figure 2:**
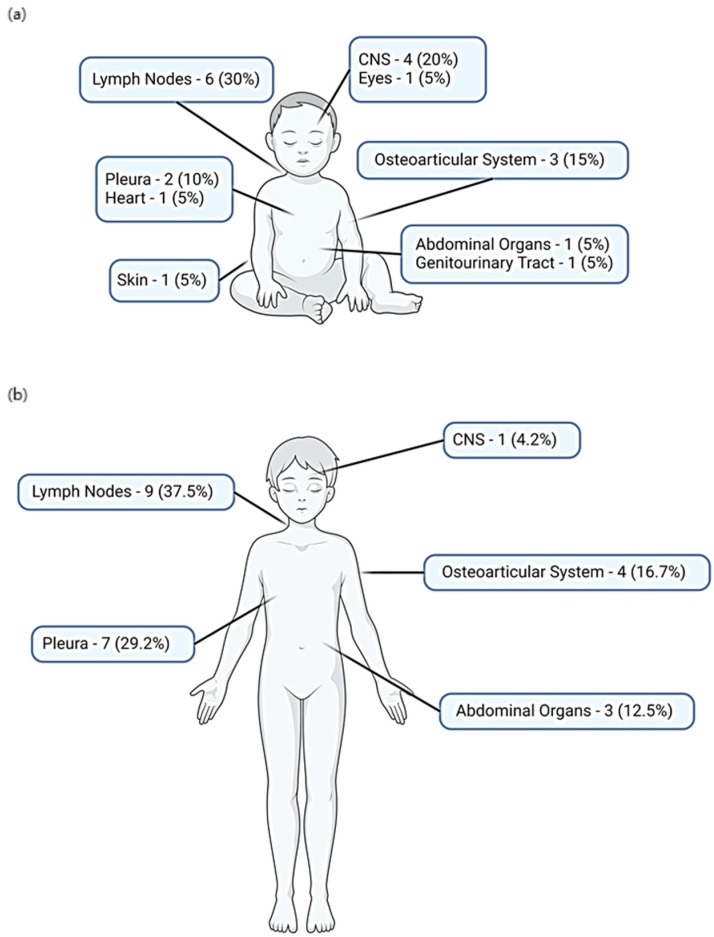
Extrapulmonary sites affected: (**a**) sites affected in children aged ≤5 years old (20/131 extrapulmonary tuberculosis cases); (**b**) sites affected in children aged >5 years old (24/140 extrapulmonary tuberculosis cases). Created with Biorender.com.

The predominant form of TB disease was pulmonary TB (PTB), accounting for 227 cases (83.76%). In 24 cases (8.86%), the disease exhibited involvement in both pulmonary and extrapulmonary sites, while only extrapulmonary sites were affected in 20 cases (7.38%). Among extrapulmonary TB (EPTB) cases, tubercular lymphadenitis was the most common, manifesting in 15 cases (15/44, 34.09%). Other sites involved included the pleura in 9 cases (9/44, 20.45%), the osteoarticular system in 7 cases (7/44, 15.91%), the central nervous system (CNS) in 5 cases (5/44, 11.36%), and abdominal organs in 4 cases (4/44, 9.09%). Notably, children presenting with tubercular meningitis were mostly aged ≤5 years old (4/5, 80.00%). The extrapulmonary sites affected in children ≤5 years old and >5 years old are described in Figure 2. Children from Asia had a higher proportion of EPTB cases (14/45; 31.11%; *p* = 0.013), compared to the rest of the geographical areas (Figure 1).

Within the study population, 58 children (58/271, 21.40%) exhibited drug susceptibility in drug-susceptibility testing (DST), while 9 cases (9/271, 3.32%) were found to be affected by drug-resistant strains. The drug-susceptibility pattern remained unidentified in 204 cases (204/271, 75.28%). Notably, among children diagnosed between 2006 and 2013, 17/154 (11.04%) had DST results, while between 2014 and 2022, they were 50/117 (42.73%, *p* < 0.001). 

**Figure 3:**
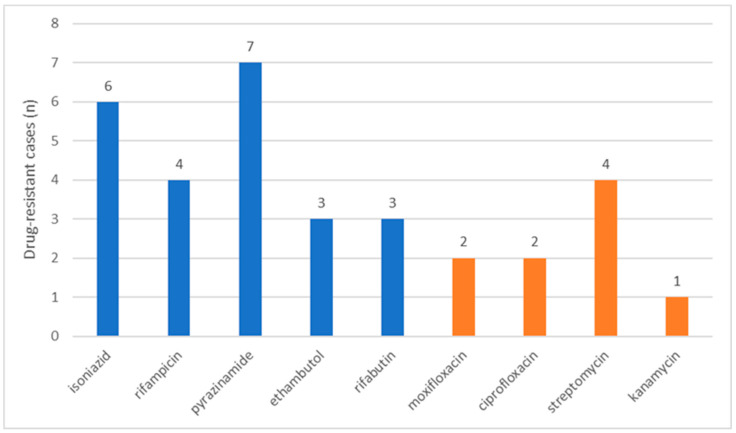
Drugs to which the study cases were found to be resistant. The number of cases found to be resistant is indicated for each drug. Blue columns correspond to drugs administered in first-line regimens. *Orange columns correspond to second-line drugs*.

The resistant cases included 2 MDR-TB cases (22.22%), 1 preXDR-TB case (11.11%), and 1 XDR-TB case (11.11%). The drugs to which they exhibited resistance, and the number of cases resistant to each single drug are detailed in Figure 3. Notably, a higher proportion of resistant cases originated from Asia (4/9; 44.44%; *p* = 0.045), as depicted in Table A3, detailing the distribution of resistant and non-resistant cases. Furthermore, the proportion of resistant cases was evenly distributed between PTB and EPTB cases, as well as among children aged ≤5 years and >5 years.

### 3.3. Diagnosis

Diagnosis of TB infection was conducted using TST and/or IGRA testing. Children with a diagnosis of infection numbered 249 (91.88%). Specifically, 209 (77.12%) were found to be positive to TST, and 224 (82.66%) to IGRAs.

The diagnosis of disease was microbiologically confirmed in 123 cases (45.39%), using culture, microscopy, and PCR or GeneXpert tests. Various specimens were collected:

- Gastric aspirate: collected in 182 cases (67.16%) and associated with positive results in 81 cases (81/182, 44.50%).
- Stool: collected in 81 cases (29.89%) and associated with positive results in 17 cases (17/81, 20.99%).
- Sputum: collected in 70 cases (25.83%) and associated with positive results in 32 cases (32/70, 45.71%).
- Biopsy: collected in 11 cases (4.06%) and associated with positive results in 9 cases (9/11, 81.82%).
- Broncho-alveolar lavage fluid: collected in 11 cases (4.06%) and associated with positive results in 5 cases (5/11, 45.45%).
- Cerebrospinal fluid: collected in 9 cases (3.32%) and associated with positive results in 1 case (1/9, 11.11%).
- Pleural fluid: collected in 7 cases (2.58%) and associated with positive results in 1 case (1/7, 14.29%).

Globally, culture resulted positive in 105 cases (38.74%), microscopy in 36 cases (13.28%), PCR in 32 cases (11.81%), and GeneXpert tests in 42 cases (15.50%).

Children were diagnosed with TBM in 5 cases, determined by clinical examination and neuroimaging. The diagnosis was microbiologically confirmed in 4 cases (80.00%): specimens associated with positive results were cerebrospinal fluid in 1 case, sputum in 1 case, and gastric aspirate in 2 cases. Neuroimaging and microbiological tests were performed within 3 days of hospitalisation.

### 3.4. Treatment

The treatment regimens only comprised first-line drugs for the majority of patients (204/271; 75.28%). However, 53 cases (19.56%) were also administered second-line drugs. Second-line drugs were more prominently utilised in cases exhibiting drug resistance (7/9; 77.78%), compared to non-resistant cases (46/262; 17.56%). The second-line drugs used in resistant patients (Table 2 and Table A4) included amikacin (5/9, 55.56%, *p* = 0.002), cycloserine (6/9, 66.67%, *p* < 0.001), linezolid (5/9, 55.56%, *p* < 0.001), moxifloxacin (4/9, 44.44%, *p* = 0.020), levofloxacin (2/9, 22.22%, *p* = 0.014), and para-aminosalicylic acid (2/9, 22.22%, *p* = 0.009). 

Two children with DR-TB did not receive second-line drugs: both the patients tested resistant only to pyrazinamide and were treated with the three first-line drugs: isoniazid, rifampicin, and ethambutol.

Children without DST results were infected by a DR-TB contact-case in 14 cases. These patients were treated with first-line drugs in 5 cases (35.71%), and with second-line drugs in 9 cases (64.29%). Moxifloxacin was administered in 9 cases (64.29%), amikacin in 7 cases (50.00%) linezolid in 6 cases (42.86%), cycloserine in 5 cases (35.71%), and para-aminosalicylic acid (PAS) in 3 cases (21.43%).

Similarly, within the population diagnosed with EPTB, second-line drugs were employed in 20 out of 44 cases (45.45%), compared to the population diagnosed with PTB, where second-line drugs were used in 33 out of 227 cases (14.54%). Moxifloxacin (13/44; 29.55%, *p* = 0.001) and cycloserine (8/44, 18.18%, *p* = 0.004) emerged as the drugs that statistically were the most administered in EPTB cases. Other second-line drugs prescribed included amikacin, linezolid, levofloxacin, ciprofloxacin, clarithromycin, and streptomycin (Table 3). Among these patients (Table 4), 3 were DR-TB cases (1 ocular TB, 1 cardiac TB, 1 tubercular lymphadenitis), 5 were DS-TB cases (2 TBM, 1 osteoarticular TB, 1 tubercular lymphadenitis, 1 pleural TB), and 9 did not have DST results (3 TBM, 3 pleural TB, 2 osteoarticular TB, 2 tubercular lymphadenitis, 1 cardiac TB, 1 abdominal TB).

Children with TBM were treated with second-line drugs: moxifloxacin in 4 cases (80.00%), cycloserine in 3 cases (60.00%), amikacin in 3 cases (60.00%).

Finally, 20 non-DR PTB cases were treated with second-line drugs.

A total of 971 drugs were prescribed. Among these, 890 (91.66%) were administered for the entire recommended duration of the regimen. In the other cases, drugs were suspended for various reasons: 42 drugs (4.33%) due to adverse events; 9 (0.93%) after the DST results became available, due to patient resistance or index case resistance; and 3 (0.31%) due to treatment adherence issues. Additionally, 2 drugs (0.21%) were suspended due to difficulties in intravenous administration in one patient. Linezolid was suspended in 1 case after DST showed complete susceptibility to first-line drugs. 

**Figure 4:**
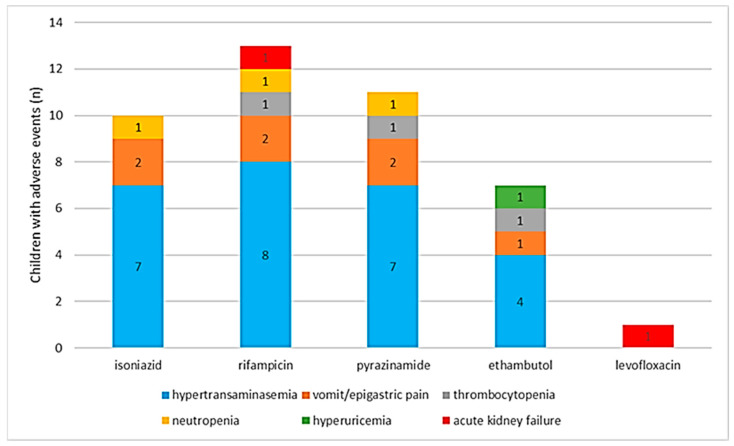
Adverse events associated with administered drugs, and number of children presenting adverse events.

Adverse events presented in 13 children (Figure 4). Hypertransaminasemia was associated with the suspension of 26 drugs (26/42; 65.00%) in children who were administered isoniazid, rifampicin, pyrazinamide, and ethambutol. Notably, these drugs were administered simultaneously in the majority of these cases; in fact, this adverse event presented in 10 children. Isoniazid, rifampicin, pyrazinamide, and ethambutol were suspended a total of 5 times (5/42, 12.5%) in 2 children, due to vomiting and epigastric pain. One child had rifampicin, pyrazinamide, and ethambutol (3/42, 7.50%) suspended due to thrombocytopenia, and another patient suspended isoniazid, rifampicin, and pyrazinamide (3/42, 7.50%) due to the emergence of neutropenia. Ethambutol was suspended in 1 case (1/42, 2.50%) due to hyperuricemia. One patient suffered acute kidney injury after the administration of therapy; consequently, rifampicin and levofloxacin (2/42, 5.00%) were suspended. 

After these children experienced adverse events, 9 cases (69.23%) re-initiated therapies (after a median discontinuation period of 4 weeks) and completed the treatment regimen; 2 cases (15.38%) re-initiated therapies (after a median discontinuation period of 3 weeks) and experienced adverse events again; 1 case (7.69%) suspended ethambutol and completed the treatment regimen with rifampicin, isoniazid, and pyrazinamide; 1 case (7.69%) experienced acute kidney injury but managed to complete therapy through a reduction in the dosages of rifampicin and levofloxacin.

### 3.5. Outcome

All children included in the study survived; 267 cases were considered cured (267/271, 98.52%) and 4 cured with sequelae (4/271, 1.48%). Among these cases, one child had EPTB and MDR-TB (1/4, 25.00%): this case was the only one that affected eye, resulting in monocular blindness. The other 3 cases had PTB (3/4, 75.00%), associated with sequelae affecting the lungs and respiratory function.

### 3.6. Factors Associated with Drug-Resistant Tuberculosis

Univariate analysis (Table 5) was used to evaluate factors associated with DR-TB. 

Notably, the origin of children and/or parents was significantly associated with DR-TB, particularly Asia (OR: 10.927, *p* = 0.035) and South-Central America (OR: 10.182, *p* = 0.048). Factors that were found to be irrelevant included the sex and age of children, the localisation of the disease, and the calendar period.

### 3.7. Factors Associated with Extrapulmonary Tuberculosis

Evaluating data with univariate analysis, factors associated with EPTB (Table 6) included the origin of children and/or parents from Asia (OR: 2.710, *p* = 0.039), the presence of symptoms (OR: 13.143, *p* < 0.001), and the calendar period 2016–2020 (OR: 4.767, *p* = 0.003). The gender and age of children and drug susceptibility of the disease were found to be irrelevant. Multivariate analysis (Table 6) was conducted, focussing on the risk factors for EPTB emerging from the univariate analysis, considering origin from Asia (OR: 2.758, *p* = 0.041) and the calendar period 2016–2020 (OR: 5.088, *p* = 0.003), which were confirmed as factors associated with EPTB.

### 3.8. Factors Associated with Use of Second-Line Drugs

Finally, treatment management was evaluated with univariate analysis (Table 7). Factors significantly associated with the administration of second-line drugs included the presence of symptoms (OR: 2.961, *p* = 0.001), having EPTB (OR: 4.899, *p* < 0.001), having the diagnosis confirmed with microbiological testing (OR: 2.472, *p* = 0.010), and the calendar period 2011–2015 (OR: 5.268, *p* < 0.001). However, the gender and age of children and the country of origin did not influence the administration of second-line drugs. These factors, found to be relevant in the univariate analysis, were evaluated in multivariate analysis (Table 7), confirming that having EPTB (OR: 4.104, *p* = 0.002), having the diagnosis confirmed by microbiological testing (OR: 2.484, *p* = 0.021), and the calendar period 2011–2015 (OR: 4.737, *p* = 0.002) were associated with the use of second-line drugs. However, the presence of symptoms was not confirmed as a significant factor in the multivariate analysis (OR: 1.749, *p* = 0.153).

## 4. Discussion

Children with active TB were evaluated for their characteristics (focussing on cases of EPTB and DR-TB) and the administrated therapies. Originating from Asia was associated with a higher risk of presenting with EPTB or DR-TB, while other characteristics, including age and gender, did not influence the distribution of these cases. The management of second-line drugs was mainly influenced by disease characteristics, such as the presence of EPTB or DR-TB. Results of DST were not available for the majority of children, and therapies had to be managed empirically, but the proportion of children with DST results increased over the years. Adverse events were primarily associated with the administration of first-line drugs, while second-line drugs were suspended in one patient only. All children were considered cured, and only a minority had sequelae despite the treatment.

Age did not appear to influence the distribution of cases, although other studies have reported a higher incidence rate in younger children [24]. However, younger age was associated with tuberculous meningitis; a similar result was found in a German study, where children under 5 years of age presented a higher percentage of tuberculous meningitis cases [25], and in a 2009 retrospective cohort study conducted in South Africa, where the median age of meningitis cases was between 2 and 4 years of age [26]. The fact that tuberculous meningitis mostly affects younger children, associated with the severity of the condition, makes its management challenging, even though it represents only a minority of TB cases.

Originating from Asia emerged as a risk factor for DR-TB, consistent with the WHO list of high-burden countries for RR/MDR-TB, where 15 out of 29 countries are Asian [2]. This finding is also supported by publications, including Liu et al. and White et al., who evaluated TB characteristics in immigrants, and mentioned China, Vietnam, India, and the Philippines as the main countries of origin for cases presenting with DR-TB [27,28]. The higher risk of presenting with EPTB among Asian people was similarly found in other studies, in Italy by Scotto et al., in which EPTB in patients from Asia were 34/60 [29], and in Spain by Luque et al., in which EPTB in patients from Asia were 134/347 [30].

The diagnosis was mainly accomplished after screening in children ≤ 5 years of age, while it was due to the presence of symptoms in children > 5 years of age. This could be related to the fact that PTB is associated with non-specific or absent symptoms in younger children, while older children may develop a more typical clinical pattern.

The difficulty in obtaining DST results in the paediatric population is a well-known problem [4], and it complicated TB management in this study as well. For this reason, the majority of children were managed with empirical treatment. It should be noted that DST results were increasingly obtained over the years, so that the proportion of children without DST results to children with results improved from 2006–2013 to 2014–2022. This is probably due to the introduction of new laboratory tests, such as GeneXpert MTB/RIF and GeneXpert Ultra. Even though variable in different studies, the sensitivity was generally high, from 67.6% to 97.5%, with pooled value of 90.0% [31].

Children with drug-resistant TB constituted a minority of cases. Excluding first-line drugs, streptomycin showed the highest resistance rate. Although the limited number of cases does not allow a definitive conclusion to be drawn, the high proportion of streptomycin-resistant strains is well documented in other studies [32,33]. The use of streptomycin in first-line regimens during the past century has led to the development of streptomycin resistance [34]. Even though it is no longer included in first-line regimens in current guidelines, streptomycin resistance continues to be detected frequently, complicating TB management [35].

Predictably, resistant cases were treated using second-line drugs. Amikacin, cycloserine, linezolid, moxifloxacin, levofloxacin, and PAS were found to be the most administered drugs in these cases. This is consistent with recommendations [4], and with DST results for the children in the study. Second-line drugs were also administered in children with non-resistant TB. This could be a consequence of the fact that most children could not be evaluated with DST and that during empirical treatment, second-line drugs could have been used because of an unsatisfactory response to first-line drugs. 

Second-line drugs administration was also associated with EPTB diagnosis, even though guidelines state that drug-susceptible cases should be treated with regimens containing first-line drugs [4]. These drugs were mainly used when TB affected challenging and difficult-to-treat localisations, including the CNS, bone, abdominal organs, the heart, and eye. Unsatisfactory results from first-line regimens and the concern of sequelae probably led to this result. Considering the site of disease, second-line drugs that were significantly associated with EPTB diagnosis were moxifloxacin and cycloserine, both also showing good penetration in sites such as CNS and bone [4]. Guidelines recommend first-line drugs for EPTB treatment [4], but the benefit to associate them with second-line drugs when treating sites that are difficult to penetrate, such as the CNS, is supported by other studies [36]. It is to be noted that second-line drugs administration should be as limited as possible to confirmed and probable DR-TB cases, as recommended by guidelines [4,16]. 

The use of second-line drugs was also influenced by the year in which these patients were treated, in particular in the period 2011–2015. This is probably associated with an increasingly conservative administration of second-line drugs in subsequent years.

The minority of children presented adverse events. Among these, hypertransaminasemia, vomiting, and epigastric pain were the most frequent, and were attributable to hepatotoxicity. Other adverse events were mostly observed in isolated cases. The association between anti-TB drugs and hepatotoxicity is known and has been analysed in numerous studies [37,38], but the fact that few children experienced it is consistent with Tersigni et al., who observed a lower incidence rate of hypertransaminasemia in the paediatric population [39] compared to the incidence rate in the general population [38]. Although the majority of studies associate second-line drugs with adverse events [40,41], only one second-line drug was suspended due to acute kidney injury, suggesting that second-line drugs were well tolerated by the children in the study. 

New antitubercular drugs bedaquiline and delamanid are recommended for DR-TB treatment in the most recent guidelines [4,16]. They are associated with favourable outcomes [42], but this study could not evaluate their efficacy and association with adverse events, because bedaquiline and delamanid were not used in our population. 

Limitations of our study included the absence of a long-term follow-up, which might have led to an overestimation of positive outcomes due to the potential missed detection of relapses and sequelae. There may also have been under-reporting of adverse events by patients and their families. Additionally, not all cases had a confirmed diagnosis. Considering the 17-year length of the study period, the number of cases is rather small (consistent with the incidence of TB in the geographical area where Meyer Hospital is located). Moreover, the low prevalence of drug-resistant cases limited the possibility of conducting strong statistical analyses. The study does not evaluate the recently approved drugs bedaquiline and delamanid. Finally, some missing data did not allow for an understanding of the reasons for all the choices that were made.

## 5. Conclusions

Paediatric TB presents several challenges, particularly in the diagnosis of the disease and obtaining DST results. The introduction of GeneXpert tests has significantly improved these issues. The geographic origin was confirmed a relevant factor: children from Asia had a higher risk of EPTB and DR-TB. In our population, anti-tubercular treatments are associated with favourable outcomes in almost all patients. Study children with EPTB were more likely to receive second-line drugs. While the administration of second-line drugs should be limited primarily to documented or suspected DR-TB cases, it is possible that these drugs may also be beneficial in selected EPTB cases that do not respond satisfactorily to recommended first-line treatments. 

## Figures and Tables

**Table 1:** Characteristics of the study population (n = 271), classified by age.

Characteristics of Children in the Study	Children ≤ 5 Years Old	Children > 5 Years Old	Total	*p*
	n = 131 (%)	n = 140 (%)	n = 271 (%)	
Male	68 (51.91)	74 (52.86)	142 (52.40)	0.972
Female	63 (48.09)	66 (47.14)	129 (47.60)	
Born in Italy to Italian Parents	37 (28.24)	26 (18.57)	63 (23.25)	0.082
Born in Italy to Foreign Parents	65 (49.62)	33 (23.57)	98 (36.16)	<0.001
Born Abroad	29 (22.14)	81 (57.86)	110 (40.59)	<0.001
Diagnosis after Contact with a Case	86 (65.65)	58 (41.43)	144 (53.14)	<0.001
Diagnosis during Screening ^1^	12 (9.16)	13 (9.29)	25 (9.22)	0.845
Diagnosis in Children with Symptoms	31 (23.66)	68 (48.57)	99 (36.53)	<0.001
Unknown	2 (1.53)	1 (0.71)	3 (1.11)	-
BCG ^2^ Vaccinated	17 (12.98)	26 (18.57)	43 (15.87)	0.274
Not BCG Vaccinated	114 (87.02)	114 (81.43)	228 (84.13)	
Pulmonary Tuberculosis	111 (84.73)	116 (82.86)	227 (83.76)	0.800
Extrapulmonary Tuberculosis ^3^	20 (15.27)	24 (17.14)	44 (16.24)	
Positive TST ^4^/QuantiFERON	118 (90.08)	131 (93.57)	249 (91.88)	0.494
Negative TST/QuantiFERON	9 (6.87)	6 (4.29)	15 (5.53)	
Unknown	4 (3.05)	3 (2.14)	7 (2.59)	-
Microbiologically ^5^ Confirmed	60 (45.80)	63 (45.00)	123 (45.39)	0.677
Not Microbiologically Confirmed	53 (40.46)	48 (34.29)	101 (37.27)	
Unknown/Absent Result	18 (13.74)	29 (20.71)	47 (17.34)	-
Positive PCR ^6^	33 (25.19)	44 (31.42)	77 (28.41)	0.144
Negative PCR	78 (59.54)	66 (47.14)	144 (53.14)	
Unknown/Absent Result	20 (15.27)	30 (21.43)	50 (18.45)	-
Positive Microscopy	13 (9.92)	23 (16.43)	36 (13.28)	0.089
Negative Microscopy	99 (75.57)	87 (62.14)	186 (68.63)	
Unknown/Absent Result	19 (14.50)	30 (21.43)	49 (18.08)	-
Positive Culture	50 (38.17)	56 (40.00)	106 (39.11)	0.426
Negative Culture	63 (48.09)	55 (39.29)	118 (43.54)	
Unknown/Absent Result	18 (38.17)	29 (28.71)	47 (17.34)	-
Drug-Resistant Tuberculosis	4 (3.05)	5 (3.57)	9 (3.32)	1.000
Drug-Susceptible Tuberculosis	27 (20.61)	31 (22.14)	58 (21.40)	0.874
Unknown Drug-Susceptibility Pattern	100 (76.34)	104 (74.29)	204 (75.28)	0.802
Treated with First-Line Drugs	95 (72.52)	109 (77.86)	204 (75.28)	0.164
Treated with Second-Line Drugs	31 (23.66)	22 (15.71)	53 (19.56)	
Unknown Therapy	5 (3.82)	9 (6.43)	14 (5.2)	-
Children Cured	129 (98.47)	138 (98.57)	267 (98.52)	1.000
Children with Sequelae	2 (1.53)	2 (1.43)	4 (1.48)	

^1^ Screening for immigrated/adopted children. ^2^ BCG: Bacillus Calmette–Guerin. ^3^ Cases exclusively affecting extrapulmonary sites and cases affecting both lungs and extrapulmonary sites were included in this group. ^4^ TST: Tuberculin Skin Test. ^5^ Diagnosis achieved through PCR/microscopy/culture. ^6^ Polymerase chain reaction.

**Table 2:** Second-line drugs administered to drug-resistant cases, drug-susceptible cases, and children without drug susceptibility testing results.

Second-Line Drugs	Drug-Resistant TB ^1^ n = 9 (%)	Drug-Susceptible TB n = 58 (%)	Children without DST ^2^ Results n = 204 (%)	*p* ^3^
Amikacin	5 (55.56)	9 (15.52)	19 (9.31)	0.002
Cycloserine	6 (66.67)	2 (3.45)	11 (5.39)	<0.001
Linezolid	5 (55.56)	2 (3.45)	8 (3.92)	<0.001
Moxifloxacin	4 (44.44)	8 (13.79)	24 (11.76)	0.020
Levofloxacin	2 (22.22)	3 (5.17)	1 (0.49)	0.014
Ciprofloxacin	0 (0.00)	0 (0.00)	1 (0.49)	1.000
Clarithromycin	0 (0.00)	0 (0.00)	1 (0.49)	1.000
Streptomycin	0 (0.00)	0 (0.00)	1 (0.49)	1.000
Para-aminosalicylic acid	2 (22.22)	0 (0.00)	3 (1.47)	0.009

^1^ TB: tuberculosis. ^2^ DST: drug-susceptibility testing. ^3^ Calculating P-value, “drug-susceptible TB” and “children without DST results” were considered as a single group and compared to “drug-resistant TB”.

**Table 3:** Second-line drugs administered to pulmonary tuberculosis cases and extrapulmonary tuberculosis cases.

Second-Line Drugs	Pulmonary Tuberculosis Cases n = 227 (%)	Extrapulmonary Tuberculosis Cases n = 44 (%)	*p*
Amikacin	25 (11.01)	8 (18.18)	0.281
Cycloserine	11 (4.85)	8 (18.18)	0.004
Linezolid	12 (5.29)	3 (6.82)	0.717
Moxifloxacin	23 (10.13)	13 (29.55)	0.001
Levofloxacin	4 (1.76)	2 (4.55)	0.252
Ciprofloxacin	0 (0.00)	1 (2.27)	1.000
Clarithromycin	0 (0.00)	1 (2.27)	1.000
Streptomycin	0 (0.00)	1 (2.27)	1.000
Para-aminosalicylic acid	5 (2.20)	0 (0.00)	1.000

**Table 4:** Drug susceptibility of pulmonary and extrapulmonary tuberculosis cases.

Drug Susceptibility	Pulmonary Tuberculosis Cases n = 227 (%)	Extrapulmonary Tuberculosis Cases n = 44 (%)	*p*
Drug-resistant tuberculosis	6 (2.65)	3 (6.82)	0.165
Drug-susceptible tuberculosis	42 (18.50)	16 (36.36)	0.015
Children without DST ^1^ results	179 (78.85)	25 (56.82)	0.004

^1^ DST: drug-susceptibility testing.

**Table 5:** Univariate analysis for factors associated with drug-resistant tuberculosis.

		Univariate Analysis		
Study Population Characteristics	n/N *	OR ^1^	95% CI ^2^	*p*
Male	3/142	1.000		
Female	6/129	2.260	0.553–9.230	0.256
≤5 years	4/131	0.850	0.223–3.238	0.812
>5 years	5/140	1.000		
Europe	1/113	1.000		
Africa	1/72	1.577	0.097–25.624	0.749
Asia	4/44	10.927	1.186–100.639	0.035
South-Central America	3/36	10.182	1.025–101.173	0.048
Pulmonary tuberculosis	6/227	0.371	0.089–1.543	0.173
Extrapulmonary tuberculosis	3/44	1.000		
Diagnosed in 2006–2015	4/178	1.000		
Diagnosed in 2016–2022	5/93	2.472	0.647–9.435	0.186

^1^ OR: odds ratio. ^2^ CI: confidence interval; * n/N: number of drug-resistant cases/number of total cases.

**Table 6:** Univariate and multivariate analysis for factors associated with extrapulmonary tuberculosis.

			Univariate Analysis			Multivariate Analysis	
Study Population Characteristics	n/N *	OR ^1^	95% CI ^2^	*p*	OR	95% CI	*p*
Male	27/142	1.000					
Female	17/129	0.646	0.334–1.251	0.195			
≤5 years	20/131	0.871	0.456–1.665	0.676			
>5 years	24/140	1.000					
Italy	9/63	1.000					
Eastern Europe	5/48	0.698	0.218–2.235	0.544	0.675	0.204–2.231	0.520
Africa	11/72	1.082	0.417–2.809	0.871	1.064	0.401–2.824	0.900
Asia	14/45	2.710	1.051–6.983	0.039	2.758	1.041–7.305	0.041
South-Central America	5/36	0.968	0.298–3.146	0.957	1.150	0.341–3.875	0.822
Diagnosis after contact	6/144	1.000					
Diagnosis during screening ^3^	2/25	2.000	0.380–10.519	0.413			
Diagnosed with symptoms	36/99	13.143	5.268–32.788	<0.001			
DR-TB ^4^ cases	3/9	2.695	0.648–11.211	0.173			
Non DR-TB cases	41/262	1.000					
Diagnosed in 2006–2010	5/74	1.000					
Diagnosed in 2011–2015	17/104	2.697	0.947–7.675	0.063	2.670	0.920–7.748	0.071
Diagnosed in 2016–2020	19/74	4.767	1.673–13.581	0.003	5.088	1.750–14.795	0.003
Diagnosed in 2021–2022	3/19	2.587	0.029–0.180	0.224	2.438	0.510–11.653	0.264

^1^ OR: odds ratio. ^2^ CI: confidence interval. ^3^ Screening for immigrated/adopted children. ^4^ DR-TB: drug-resistant tuberculosis. * n/N: number of extrapulmonary cases/number of total cases.

**Table 7:** Univariate and multivariate analyses for factors associated with second-line drugs administration.

Study Population Characteristics	n/N *	OR ^1^	95% CI ^2^	*p*	OR	95% CI	*p*
Male	29/142	1.000					
Female	24/129	0.891	0.487–1.627	0.706			
≤5 years	31/131	1.663	0.905–3.054	0.101			
>5 years	22/140	1.000					
Italy	10/63	1.000					
Eastern Europe	10/48	1.395	0.528–3.681	0.502			
Africa	11/72	0.956	0.376–2.427	0.924			
Asia	13/45	2.153	0.846–5.478	0.108			
South-Central America	7/36	1.279	0.440–3.717	0.651			
Diagnosis after contact	20/144	1.000					
Diagnosis during screening ^3^	0/25						
Diagnosed with symptoms	32/99	2.961	1.573–5.575	0.001	1.749	0.813–3.761	0.153
Pulmonary tuberculosis	33/227	1.000					
Extrapulmonary tuberculosis ^4^	20/44	4.899	2.436–9.854	<0.001	4.104	1.691–9.962	0.002
Microbiologically confirmed ^5^	35/123	2.472	1.243–4.913	0.010	2.484	1.146–5.384	0.021
Not microbiologically confirmed	14/101	1.000					
Diagnosed in 2006–2010	6/74	1.000					
Diagnosed in 2011–2015	33/104	5.268	2.076–13.368	<0.001	4.737	1.764–12.715	0.002
Diagnosed in 2016–2020	10/74	1.771	0.608–5.153	0.294	0.909	0.282–2.931	0.874
Diagnosed in 2021–2022	4/19	3.022	0.758–12.051	0.117	1.717	0.387–7.611	0.477

^1^ OR: odds ratio. ^2^ CI: confidence interval. ^3^ Screening for immigrated/adopted children. ^4^ Cases exclusively affecting extrapulmonary sites and cases affecting both lungs and extrapulmonary sites were included in this group. ^5^ Diagnosis achieved through PCR/microscopy/culture. * n/N: number of cases associated with second-line drugs administration/number of total cases.

## Data Availability

Data are contained within the article.

## Appendix A

**Table A1:** Characteristics of the study population (n = 271), classified by disease localisation.

Characteristics of Children in the Study	Children with PTB ^1^ N = 227 (%)	Children with EPTB ^2^ N = 44 (%)	*p*
Male	115 (50.66)	27 (61.36)	0.256
Female	112 (49.34)	17 (38.64)	
[0–1) years	5 (2.20)	1 (1.72)	1.000
[1–3) years	23 (10.13)	6 (13.64)	0.673
[3–5) years	69 (30.40)	14 (31.82)	0.993
[5–13) years	91 (40.09)	14 (31.82)	0.389
≥13 years	39 (17.18)	10 (22.73)	0.509
Born in Italy to Italian Parents	54 (23.79)	9 (20.45)	0.776
Born in Italy to Foreign Parents	84 (37.00)	14 (31.82)	0.628
Born Abroad	89 (39.21)	21 (47.73)	0.376
Diagnosis after Contact with a Case	138 (60.79)	6 (13.64)	<0.001
Diagnosis during Screening ^3^	23 (10.13)	2 (4.55)	0.393
Diagnosis in Children with Symptoms	63 (27.75)	36 (81.82)	<0.001
Unknown	3 (1.32)	0 (0.00)	-
BCG ^4^ Vaccinated	36 (15.86)	7 (15.91)	0.828
Not BCG Vaccinated	191 (84.14)	37 (84.09)	
Positive TST ^5^/QuantiFERON	209 (92.07)	40 (90.91)	0.479
Negative TST/QuantiFERON	14 (6.17)	1 (2.27)	
Unknown/Absent Result	4 (1.76)	3 (6.82)	-
Microbiologically ^6^ Confirmed	98 (43.17)	25 (56.82)	0.084
Not Microbiologically Confirmed	90 (39.65)	11 (25.00)	
Unknown/Absent Result	39 (17.18)	8 (18.18)	-
Positive PCR ^7^	58 (25.55)	19 (43.18)	0.015
Negative PCR	128 (56.39)	16 (36.36)	
Unknown/Absent Result	41 (18.06)	9 (20.45)	-
Positive Microscopy	28 (12.33)	8 (18.18)	0.412
Negative Microscopy	158 (69.60)	28 (63.64)	
Unknown/Absent Result	41 (18.06)	8 (18.18)	-
Positive Culture	84 (37.00)	22 (50.00)	0.104
Negative Culture	104 (45.81)	14 (31.82)	
Unknown/Absent Result	39 (17.18)	8 (18.18)	-
Drug-Resistant Tuberculosis	6 (2.64)	3 (6.82)	0.165
Drug-Susceptible Tuberculosis	42 (18.51)	16 (36.36)	0.015
Unknown Drug-Susceptibility Pattern	179 (78.85)	25 (56.82)	0.003
Treated with First-Line Drugs	182 (80.18)	22 (50.00)	<0.001
Treated with Second-Line Drugs	33 (14.54)	20 (45.45)	
Unknown Therapy	12 (5.28)	2 (4.55)	-
Children Cured	224 (98.68)	43 (97.72)	0.510
Children with Sequelae	3 (1.32)	1 (2.28)	

^1^ PTB: pulmonary tuberculosis. ^2^ EPTB: extrapulmonary tuberculosis. ^3^ Screening for immigrated/adopted children. ^4^ BCG: Bacillus Calmette–Guerin. ^5^ TST: tuberculin skin test. ^6^ Diagnosis achieved through PCR/microscopy/culture. ^7^ Polymerase chain reaction.

**Table A2:** Characteristics of the study population (n = 271), classified by drug-susceptibility pattern.

Characteristics of Children in the Study	Drug-Resistant TB ^1^ Cases n = 9 (%)	Drug-Susceptible TB Cases n = 58 (%)	Children without DST ^2^ Results n = 204 (%)	*p* *
Male	3 (33.33)	38 (65.52)	101 (49.51)	0.317
Female	6 (66.67)	20 (34.48)	103 (50.49)	
[0–1) years	0 (0.00)	1 (1.72)	4 (1.96)	1.000
[1–3) years	1 (11.11)	8 (13.79)	20 (9.80)	1.000
[3–5) years	2 (22.22)	16 (27.59)	65 (31.86)	0.726
[5–13) years	4 (44.44)	20 (34.68)	81 (39.71)	0.738
≥13 years	2 (22.22)	13 (22.41)	34 (16.67)	0.668
Born in Italy to Italian Parents	1 (11.11)	14 (24.14)	48 (23.53)	0.690
Born in Italy to Foreign Parents	5 (55.56)	20 (34.48)	73 (35.78)	0.289
Born Abroad	3 (33.33)	24 (41.38)	83 (40.69)	0.743
Diagnosis after Contact with a Case	5 (55.56)	27 (46.55)	112 (54.90)	1.000
Diagnosis during Screening ^3^	0 (0.00)	2 (3.45)	23 (11.27)	1.000
Diagnosis in Children with Symptoms	4 (44.44)	28 (48.28)	67 (32.84)	0.730
Unknown	0 (0.00)	1 (1.72)	2 (0.98)	-
BCG ^4^ Vaccinated	1 (11.11)	4 (6.90)	38 (18.63)	1.000
Not BCG Vaccinated	8 (88.89)	54 (93.10)	166 (81.37)	
Pulmonary Tuberculosis	6 (66.67)	42 (72.41)	179 (87.75)	0.165
Extrapulmonary Tuberculosis ^5^	3 (33.33)	16 (25.79)	25 (12.25)	
Positive TST ^6^/QuantiFERON	9 (100.00)	54 (93.10)	186 (91.18)	1.000
Negative TST/QuantiFERON	0 (0.00)	2 (3.45)	13 (6.37)	
Unknown/Absent Result	0 (0.00)	2 (3.45)	5 (2.45)	-
Microbiologically ^7^ Confirmed	9 (100.00)	58 (100.00)	56 (27.45)	0.005
Not Microbiologically Confirmed	0 (0.00)	0 (0.00)	101 (49.51)	
Unknown/Absent Result	0 (0.00)	0(0.00)	47 (23.04)	-
Positive PCR	5 (55.56)	46 (79.31)	26 (12.75)	0.281
Negative PCR	4 (44.44)	12 (20.69)	128 (62.75)	
Unknown/Absent Result	0 (0.00)	0 (0.00)	50 (24.51)	-
Positive Microscopy	2 (22.22)	17 (29.31)	17(8.33)	0.642
Negative Microscopy	7 (77.78)	41 (70.69)	137 (67.16)	
Unknown/Absent Result	0 (0.00)	0 (0.00)	49 (24.02)	-
Positive Culture	9 (100.00)	48 (82.76)	49 (24.02)	0.001
Negative Culture	0 (0.00)	10 (17.24)	108 (52.94)	
Unknown/Absent Result	0 (0.00)	0 (0.00)	47 (23.04)	-
Treated with First-Line Drugs	2 (22.22)	45 (77.59)	157 (76.96)	<0.001
Treated with Second-Line Drugs	7 (77.78)	13 (22.41)	33 (16.18)	
Unknown Therapy	0 (0.00)	0 (0.00)	14 (6.86)	-
Children Cured	8 (88.89)	56 (96.55)	203 (99.51)	0.127
Children with Sequelae	1 (1.11)	2 (3.45)	1 (0.49)	

^1^ TB: tuberculosis. ^2^ DST: drug-susceptibility testing. ^3^ Screening for immigrated/adopted children. ^4^ BCG: Bacillus Calmette–Guerin. ^5^ Cases exclusively affecting extrapulmonary sites and cases affecting both lungs and extrapulmonary sites were included in this group. ^6^ TST: tuberculin skin test. ^7^ Diagnosis achieved through PCR/microscopy/culture. * Calculating *p*-value, “Drug-susceptible TB cases” and “Children without DST results” were considered as a single group and compared with “Drug-resistant TB cases”.

**Table A3:** Drug-resistant tuberculosis cases and non-drug-resistant tuberculosis cases, classified by origin.

Origin of Children in the Study	Drug-Resistant TB ^1^ Cases n = 9 (%)	Drug-Susceptible TB Cases and Children without DST ^2^ Results n = 262 (%)	*p*
Italy	0 (0.00)	63 (23.75)	0.123
Western Europe	0 (0.00)	2 (0.77)	1.000
Eastern Europe	1 (11.11)	47 (18.01)	1.000
Africa	1 (11.11)	71 (27.20)	0.453
Asia	4 (44.44)	41 (15.71)	0.045
South-Central America	3 (33.33)	33 (12.64)	0.103
Unknown	0 (0.00)	5 (1.92)	1.000

^1^ TB: tuberculosis. ^2^ DST: drug-susceptibility testing.

**Table A4:** Second-line drugs administered to drug-resistant cases, and drug-susceptible cases.

Second-Line Drugs	Drug-Resistant TB ^1^ n = 9 (%)	Drug-Susceptible TB n = 58 (%)	*p*
Amikacin	5 (55.56)	9 (15.52)	0.015
Cycloserine	6 (66.67)	2 (3.45)	0.000
Linezolid	5 (55.56)	2 (3.45)	<0.001
Moxifloxacin	4 (44.44)	8 (13.79)	0.047
Levofloxacin	2 (22.22)	3 (5.17)	0.130
Para-aminosalicylic acid	2 (22.22)	0 (0.00)	0.016

^1^ TB: tuberculosis.
